# An N-terminal extension to the hepatitis B virus core protein forms a poorly ordered trimeric spike in assembled virus-like particles

**DOI:** 10.1016/j.jsb.2014.12.006

**Published:** 2015-02

**Authors:** Richard McGonigle, Wei Boon Yap, Swee Tin Ong, Derek Gatherer, Saskia E. Bakker, Wen Siang Tan, David Bhella

**Affiliations:** a MRC-University of Glasgow Centre for Virus Research, Sir Michael Stoker Building, Garscube Campus, 464 Bearsden Road, Glasgow G61 1QH, Scotland, UK; b Department of Microbiology, Faculty of Biotechnology and Biomolecular Sciences, Universiti Putra Malaysia, 43400 Serdang, Selangor, Malaysia; c Institute of Bioscience, Universiti Putra Malaysia, 43400 UPM Serdang, Selangor, Malaysia

**Keywords:** Virus-like particle, Vaccine, Hepatitis B virus, Cryo-electron microscopy, Three-dimensional reconstruction, Local resolution

## Abstract

Virus-like particles composed of the core antigen of hepatitis B virus (HBcAg) have been shown to be an effective platform for the display of foreign epitopes in vaccine development. Heterologous sequences have been successfully inserted at both amino and carboxy termini as well as internally at the major immunodominant epitope. We used cryogenic electron microscopy (CryoEM) and three-dimensional image reconstruction to investigate the structure of VLPs assembled from an N-terminal extended HBcAg that contained a polyhistidine tag. The insert was seen to form a trimeric spike on the capsid surface that was poorly resolved, most likely owing to it being flexible. We hypothesise that the capacity of N-terminal inserts to form trimers may have application in the development of multivalent vaccines to trimeric antigens. Our analysis also highlights the value of tools for local resolution assessment in studies of partially disordered macromolecular assemblies by cryoEM.

## 1 Introduction

Vaccines play a crucial role in combating infectious diseases and have led to the eradication of one notable human pathogen: variola virus that caused smallpox. Despite many successful vaccination campaigns, major threats to human health remain for which we have no adequate vaccines. Development of new vaccines is a difficult and expensive process. Traditionally, vaccines for the prevention of viral disease consist of killed or live-attenuated virus. More recently subunit vaccines, containing the antigenic components of the target pathogen have been successfully introduced, these may comprise adjuvant-peptide complexes or particulate assemblies of antigen (Rueckert and Guzmán, 2012).

Viral structural proteins can frequently be made to assemble into structures that closely resemble authentic virus particles. These assemblies are known as virus-like particles (VLPs). As a vaccine candidate VLPs have a number of advantages over peptide based subunit vaccines: ordered, multivalent antigens are more readily recognised by the immune system, and present viral antigens in an authentic conformation, stimulating a strong B-cell response (Noad and Roy, 2003). VLP based vaccines are also safer than live attenuated ones. There is no risk of reversion to virulence such as that which has hindered poliovirus eradication (Modlin, 2010). VLP based vaccines are easier than killed vaccines to produce safely since their production does not require large scale propagation of live pathogenic viruses which may pose a hazard to production workers or a risk of accidental release. VLP based subunit vaccines are currently used to vaccinate against hepatitis B virus (HBV) and human papillomavirus (HPV).

HBV is an enveloped virus that contains a partially double stranded DNA genome synthesized via a reverse transcription step and enclosed within an icosahedral capsid. HBV vaccines consist of the hepatitis B surface antigens (HBsAg), which are the major components of the viral envelope. VLPs of HBsAg are produced in HBV chronic carriers and the first-generation vaccine was prepared by purifying these non-infectious VLPs from the blood of infected individuals (Shepard et al., 2006). The modern form of HBV vaccine contains HBsAg VLPs produced in yeast via recombinant DNA technology (Valenzuela et al., 1982).

VLPs are also capable of being assembled from the dimer of core protein (HBcAg), which is 183 amino acids (aa) in length (or 185 aa in some variants of the virus). HBcAg VLPs were the first to be proposed as epitope display particles for the development of multi-valent vaccines (Pumpens and Grens, 1999). HBcAg particles are dimorphic; they may be formed from 240 subunits to produce a *T* = 4 icosahedrally symmetric particle that is 34 nm in diameter or 180 subunits to make a 30 nm diameter particle with *T* = 3 symmetry (Crowther et al., 1994; Karpenko et al., 2000). Both forms of particle have prominent spikes on their surfaces that are four-helix bundles consisting of two anti-parallel helices donated by each monomer. The loop connecting the two helices in each monomer is the major immunodominant epitope (MIE) of the core protein. The C-terminal 43 residues of HBcAg form a basic domain that interacts with the viral genome. For heterologous VLP production this region can be deleted without hindering assembly (Gallina et al., 1989).

HBcAg can activate a B-cell response independent of T-cell activation, and confers this property to normally T-cell dependant antigens displayed on the VLP (Whitacre et al., 2009). HBcAg can also spontaneously self-assemble, in the absence of other viral proteins (Karpenko et al., 2000). Assembly has been observed in a wide range of expression systems including: mammalian (Hirschman et al., 1980), insect (Hilditch et al., 1990; Seifer et al., 1998), plant (Tsuda et al., 1998) and bacterial cells (Pasek et al., 1979; Schödel et al., 1990).

To successfully exploit the above properties requires that potential vaccine constructs be designed such that the antigen is exposed on the VLP’s surface and does not interfere with particle formation. Only 3 sites, the MIE, the N- and C-termini, allow insertion of foreign antigen while fulfilling these criteria (Ulrich et al., 1998). The most immunogenic of the three insertion sites is the MIE loop, followed by the N-terminus (Schödel et al., 1996). While it is not possible to definitively predict whether a VLP will assemble with a given insert, certain characteristics have been deduced which impact on VLP formation (Nassal et al., 2008). Factors that can disrupt VLP formation include the presence of: cysteine residues (Janssens et al., 2010), β-pleated sheets and extensive regions of hydrophobicity (Karpenko et al., 2000). Formation can also be disrupted by homomeric interactions between inserts, for example propensity to form anti-parallel dimers might disrupt core dimerization (Vogel et al., 2005). The size of the insert is important; smaller inserts are more likely to permit VLP formation, owing to reduced steric hindrance. Exceptions exist however, such as the successful insertion of GFP into the MIE loop (Kratz et al., 1999). Finally incorporating flexible linker regions between insert and core increases the probability of success. For inserts into the MIE, close proximity of the N- and C-termini also improves the chances of VLP assembly as such inserts are less likely to disrupt formation of the four-helix bundle (Nassal et al., 2008).

Here we describe the structure of VLPs produced by heterologous expression of a HBcAg construct containing a polyhistidine tag within a 37 residue N-terminal extension (His-β-L HBcAg) (Yap et al., 2009). Cryo-electron microscopy and three-dimensional reconstruction at intermediate resolution revealed the presence of a second, apparently trimeric, spike on the VLP surface. Icosahedral reconstructions were calculated for both *T* = 3 and *T* = 4 VLPs. Attempts to achieve sub-nanometre resolution in these maps were frustrated however; Fourier shell correlation analysis indicating that the structures were limited to 10 and 12 Å respectively. Two local resolution assessment algorithms were used to investigate the resolution of the maps in more detail revealing that sub-nanometre resolution was achieved in the HBcAg component of the construct while the N-terminal insert was poorly ordered. Nonetheless we hypothesise that formation of a second trimeric spike on the VLP surface may have application in the design of vaccines to elicit immunity against proteins that normally exist in a trimeric state.

## 2 Materials and methods

### 2.1 Expression of N-terminal extended HBcAg

The plasmid pHis-β-L-HBcAg was previously described (Yap et al., 2009) and the amino acid sequence of its translated product was described in (Lee et al., 2012). Briefly the construct His-β-L HBcAg comprises a 37aa region (MRGSHHHHHHGMASMTGGQQMGRDLYDDDDKDPLEFH) fused to residues 3–145 of HBcAg. The N-terminal extension contains a 6-His tag, the T7 gene 10 leader, the Xpress™ epitope, amino acid sequence of pRSET vector, two residues of β-galactosidase and a 3 residues flexible linker.

*Escherichia* *coli* cells harbouring pHis-β-L HBcAg were cultured in 1 L Luria–Bertani (LB) medium at 30 °C at 200 rpm. When the cultures reached OD_600_ 0.6–0.8, recombinant protein expression was induced with 0.5 mM isopropyl β-d-thiogalactoside (IPTG) and incubated for 16–18 h. The cells were pelleted by centrifugation at 4000×*g* for 20 min; the pellet was then resuspended in HEPES buffer (25 mM; 2 ml; pH 7.6). The cells were disrupted by ultrasonication for 10 min (20 s between pulses) then centrifuged at 30,000×*g* for 20 min. The supernatant was incubated with DNAase (10 μg/ml) at 37 °C for 1 h and then precipitated with 35% ammonium sulphate saturation. This mixture was spun at 8000×*g*, dissolved in binding buffer (20 mM sodium phosphate, 500 mM NaCl, 20 mM imidazol, pH 7.4) and dialysed at 4 °C overnight.

### 2.2 Purification of VLPs

VLPs were purified by immobilised metal affinity chromatography. A HisTrap HP column (Amersham Bioscience, Pittsburg, USA) was equilibrated with 5 column volumes (CV) of binding buffer, 10 mg of sample was loaded then washed with 10 CV of binding buffer, followed by 5 CV of washing buffer (20 mM sodium phosphate, 500 mM NaCl, pH 7.4; 100 mM imidazole) and then eluted with elution buffer [(20 mM sodium phosphate; 500 mM NaCl), pH 7.4; 500 mM imidazole] (Yap et al., 2009).

### 2.3 Cryo-electron microscopy

Purified VLP suspension (5 μl) was loaded onto freshly glow-discharged quantifoil perforated carbon support films (R2/2; Quantifoil, Jena, Germany), blotted, and plunged into liquid ethane (Adrian et al., 1984). Vitrified specimens were imaged at low temperature and under low-electron dose conditions (between 12 and 15 e/Å^2^) in a JEOL 2200 FS cryo-microscope operated at 200 kV. Specimens were held in a Gatan 626 cryo-stage cooled to 95 K. To reduce the contribution of noise to the image by inelastic scattering of electrons, energy filtered imaging was performed with a slit-width of 20 eV. Images were recorded at 150,000× magnification (corresponding to a pixel size 0.69 Å in the specimen) on a Gatan Ultrascan 4k × 4k charge-coupled device camera.

### 2.4 Icosahedral reconstruction

Micrographs were binned by a factor of two prior to analysis, giving a sample frequency of 1.39 Å/pixel. The *BSOFT* package was used to estimate defocus values for each micrograph (which ranged from 0.8 to 3.8 μm) and apply CTF correction (Heymann, 2001). Particle images were processed using *SPIDER* (Frank et al., 1996) to remove low-frequency variations in density and to exclude outlying grey levels caused by e.g. X-rays. Images were then classified into two sub-populations based on correlation with fuzzy ring models representing the *T* = 3 and *T* = 4 classes. Finally particles were centred according to the peak found in the cross-correlation function during the previous step.

Initial reconstructions were calculated using a common-lines approach implemented in a modified version of the MRC icosahedral reconstruction suite of programs (Crowther et al., 1970; Fuller et al., 1996). Subsequently origins and orientations for particles in both datasets were determined using the polar Fourier transform method implemented in *PFT2* (Baker and Cheng, 1996; Bubeck et al., 2005). To guard against reference bias, refinement was performed to a maximum resolution of 14 Å. Reconstructions were then calculated using the *EM3DR2* program. Resolution was initially evaluated by halving both data sets into equal subsets, from which two reconstructions were calculated. Comparison of these maps using the *BSOFT* program *bresolve* yielded several indices of similarity including the Fourier shell correlation. Reconstructions were visualised using *UCSF Chimera* (Pettersen et al., 2004) The threshold for contouring the isosurface was based on the calculated molecular weight of the *T* = 3 and *T* = 4 VLP structures unless stated otherwise. Segmentation was performed in *UCSF Chimera* using the *Segger* plugin (Pintilie et al., 2010).

### 2.5 Local resolution assessment

Local resolution was calculated using two distinct approaches: *ResMap* (Kucukelbir et al., 2014) and a local Fourier shell correlation analysis implemented in the *BSOFT* program *Blocres* (Cardone et al., 2013; Heymann, 2001). For *ResMap* analyses resolution assessment was performed at 0.25 Å intervals at resolutions ranging between 5 and 20 Å. *Blocres* was performed using a box size of 30 at 5 voxel intervals, to a maximum resolution of 6 Å and with a cut-off FSC of 0.5. For both methods reconstructions were calculated to the Nyquist limit of the data (e.g. 2.8 Å) and used without filtering or masking of the density. Output from these routines was then used to colour the isosurfaced representation of each reconstruction.

### 2.6 Sharpening and fitting of crystallographic data

Following local resolution evaluation, sharpening of the *T* = 3 map was performed using *EMBFACTOR* (Fernández et al., 2008). The X-ray structure for HBcAg (PDB id 1QGT) was then docked into the reconstruction (Wynne et al., 1999). 1QGT contains four chains, one for each of the quasi-equivalent positions in the *T* = 4 capsid that was solved. Each of these was fitted at the three quasi-equivalent positions within the asymmetric unit of the *T* = 3 His-β-L HBcAg VLP, using *UCSF Chimera* (Pettersen et al., 2004). To establish which chain gave the best fit, a density map was generated from the X-ray coordinates at 6 Å. The fit with the highest correlation coefficient between the reconstructed density map and X-ray derived density was selected.

### 2.7 Modelling of construct tertiary structure

To determine a high resolution model of our construct the amino acid sequence was modelled in *MOE 2007.09* (Chemical Computing Group, Montreal), the sequence was first aligned against the existing HBcAg structure determined by X-ray crystallography (Wynne et al., 1999) of the *T* = 4 capsid and then Boltzmann-weighted randomized outgap modelling was used to predict the structure of the extension (Fechteler et al., 1995; Levitt, 1992). 250 models were constructed. On completion of segment addition, the highest-scoring intermediate model was then determined by the generalised Born/volume integral (GB/VI) methodology (Labute, 2008) Each model was energetically minimised in the AMBER-99 force field (Wang et al., 2000) The models were then rated by energetic criteria and absence of atom clashes and the best three models were selected for comparison with the reconstruction in *UCSF Chimera* (Pettersen et al., 2004).

## 3 Results

### 3.1 Cryomicroscopy and 3D reconstruction reveals HBcAg particles with an additional trimeric spike

**Fig.1:**
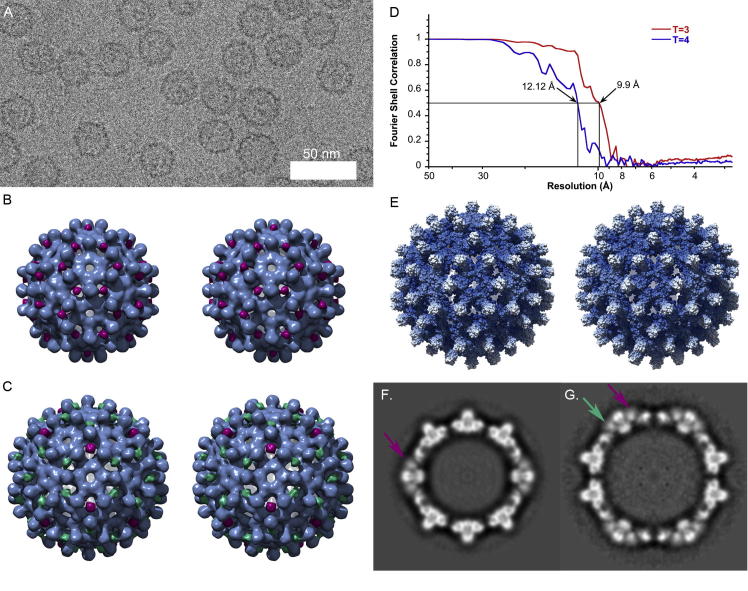
3D reconstructions of His-β-L HBcAg virus-like particles. (A) Cryomicroscopy of His-β-L HBcAg VLPs revealed two sizes 30 nm *T* = 3 and 34 nm *T* = 4. (B) The *T* = 3 structure was generated using 6048 particle images and achieved a resolution of 10 Å. (C) The *T* = 4 VLP was reconstructed using 2040 particles and achieved a resolution of 12 Å. Resolution assessment was by the FSC^0.5^ criterion (D). The general architecture of both capsids is similar to the wild type HBcAg, as demonstrated by comparison with a solvent exclusion surface representation of the *T* = 4 capsid calculated from the X-ray structure PDB ID 1QGT (E). His-β-L HBcAg particles however were seen to bear an extra spike density hypothesised to comprise a trimer of the extended N-terminus, located at sites of local threefold symmetry in the *T* = 3 VLP (magenta) and at both icosahedral (green) and local (magenta) threefold symmetry axes in the *T* = 4 particle. Wall-eyed stereo pair images are presented (B, C and E). Cross-sections through the *T* = 3 (F) and *T* = 4 (G) reconstructions show that the density in the N-terminal spikes is blurred compared to that of the HBcAg component (magenta and green arrows).

Cryomicrographs of purified His-β-L HBcAg VLPs showed the presence of two populations of differently sized particles with diameters of approximately 30 nm and 34 nm, corresponding to the expected sizes for *T* = 3 and *T* = 4 classes (Fig. 1A). The *T* = 3 form was most common (74.4%), in line with the findings of previous studies showing that truncation of the C-terminus increases the ratio of *T* = 3 versus *T* = 4 particles (Pumpens and Grens, 2001). 7046 *T* = 3 and 2419 *T* = 4 particles were extracted from 785 micrographs and processed to calculate three-dimensional reconstructions.

Following iterative refinement of particle origins and orientations for both size classes, 6048 *T* = 3 particles and 2040 *T* = 4 particles were selected and used to calculate three-dimensional reconstructions (Fig. 1B and C). FSC analysis using the 0.5 criterion indicated resolutions of 10 Å and 12 Å respectively whilst the less conservative FSC 0.143 criterion gave resolutions of 8.4 Å and 10 Å (Fig. 1D). The reconstructions are broadly consistent with the known architecture of HBcAg particles (Fig. 1E). In addition to the characteristic spikes formed by the core protein dimer however, smaller spikes of density were also seen at sites of local threefold symmetry adjacent to the larger dimeric spikes. Sixty spikes were seen in the *T* = 3 structure, coloured magenta in Fig. 1B. The location and number of these spikes is consistent with them being formed of three copies of the inserted N-terminal polypeptide (Tan et al., 2007).

In the case of the *T* = 4 structure, eighty spikes were seen; twenty at the icosahedral threefold symmetry axes (coloured magenta in Fig. 1C) and sixty arranged about the icosahedral fivefold symmetry axes, alternating with dimeric spikes and located at sites of local threefold rotational symmetry (coloured green in Fig. 1C). The novel spikes measured 2 nm from base to tip when the isosurface threshold was set to enclose the calculated molecular volume of the whole assembly. Volume measurements for each segmented spike gave 2700, 3600 and 4250 Å^3^ indicating a molecular mass between 2.2 and 3.4 kDa, however the calculated mass of a trimer of the full 37 residue insert would be considerably larger at 12.8 kDa. Central sections through the reconstructed density reveal that the spikes are rather poorly defined compared to the HBcAg component – indicating a degree of movement in the structure (Fig. 1F and G).

### 3.2 Local resolution assessment

Despite repeated attempts to extend the resolution of the maps by rigorous vetting of data quality and inclusion of significant particle numbers, efforts to derive consistent, interpretable density or evidence of secondary structure in the region attributed to the N-terminal extension were unsuccessful. To investigate the possible causes of this we evaluated the local resolution of maps using two independent approaches.

**Fig.2:**
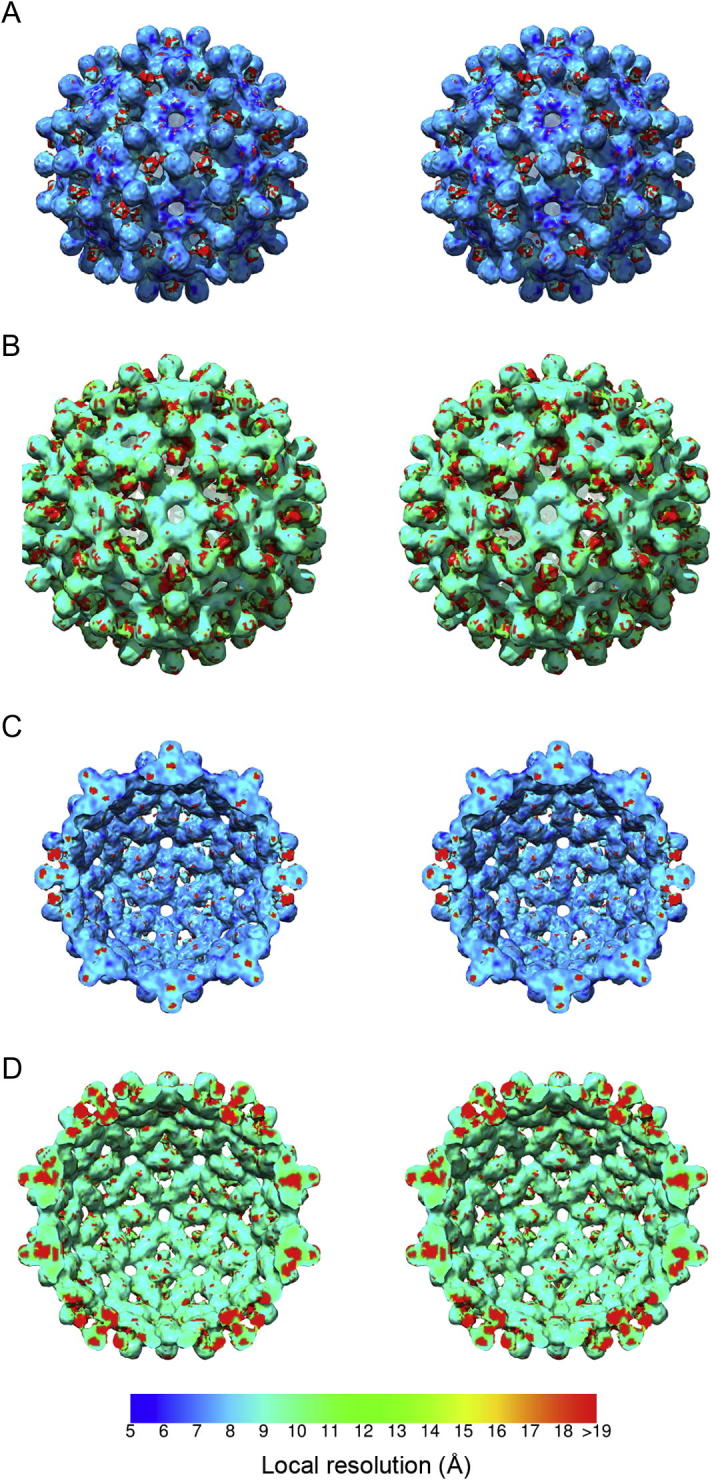
*ResMap* analysis of His-β-L HBcAg VLP reconstructions. *ResMap* was used to evaluate the local resolution of both *T* = 3 (A) and *T* = 4 (B) reconstructions. Isosurfaced representations of the reconstructions were coloured accordingly revealing that the HBcAg component was solved at higher resolution than the N-terminal extension. In the case of the *T* = 3 particle the core density achieved resolution approaching 5 Å, ranging to 8 Å in places, while the inserted polypeptide was seen to be poorer than 19 Å resolution. The *T* = 4 reconstruction was calculated from fewer particles and achieved lower resolution ranging between 8 and 10 Å in the HBcAg region and 11–>19 Å in the N-terminal extension. Interestingly both *ResMap* analyses gave resolution assessments that included small patches of poor-resolution throughout the structure, in particular within the *dimeric* spike of HBcAg, these are apparent in clipped isosurface representations (C and D).

*Resmap* analysis (Kucukelbir et al., 2014) of the *T* = 3 reconstruction indicated a mean resolution of 7.8 Å; significantly better than the estimate derived by the FSC^0.5^ method. Analysis of the *T* = 4 structure also indicated a better resolution than that estimated by Fourier shell correlation at 0.5 cutoff, the mean resolution was determined to be 10.6 Å (Fig. 2). Local resolution measurements were seen to range between 5 and 8 Å within the core regions of the *T* = 3 VLP, while in the *T* = 4 structure the HBcAg components were resolved at between 8 and 10 Å. Interestingly in both analyses the novel density that we attribute to the N-terminal extension was highlighted as having a substantially lower resolution than the HBcAg structure. For the *T* = 3 particle this region was determined to have a resolution poorer than 19 Å, likewise in the *T* = 4 structure the trimeric spike was identified as a region of low resolution.

**Fig.3:**
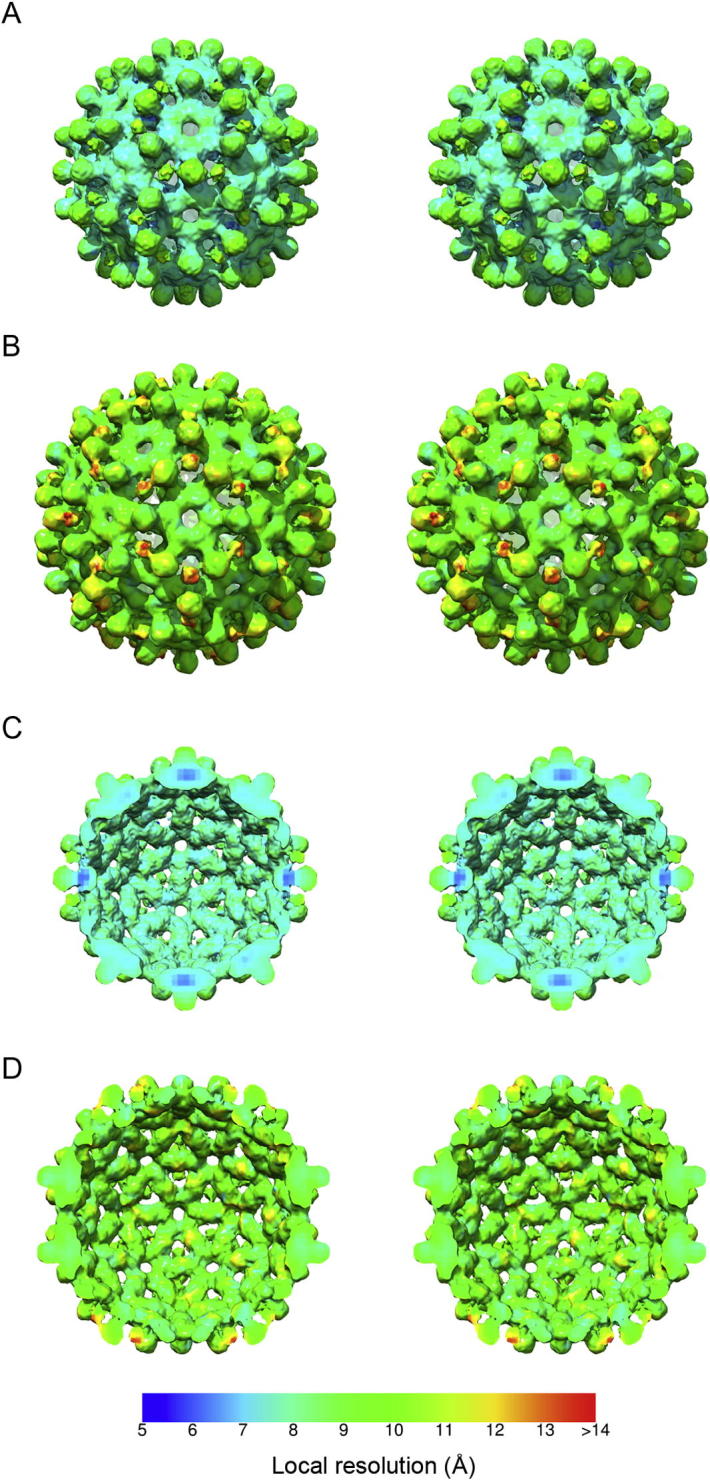
*Blocres* local resolution analysis of His-β-L HBcAg VLP reconstructions. Local FSC resolution analysis was performed using the BSOFT routine *Blocres*. The *T* = 3 (A) and *T* = 4 (B) reconstructions are presented in stereo pair view and coloured according to local resolution. For both structures, the N-terminal inserted region, has a lower resolution than the native HBcAg capsid. This finding is consistent with the *ResMap* analysis and suggests that the inserted region is more flexible than the core capsid structure. Unlike the *ResMap* analyses however the HBcAg *dimeric* spikes are assessed as being higher-resolution although the distal portions of these four-helix bundles are lower resolution than the shell of the capsid (C and D).

To confirm our findings, we evaluated local resolution within the maps using an alternative method: the Bsoft routine *Blocres* (Cardone et al., 2013). This approach calculates Fourier shell correlation values for a sliding box to yield a resolution map (Fig. 3). Supporting the results obtained with *ResMap*, *Blocres* determined that the *T* = 3 structure was resolved at between 6 and 9 Å within the HBcAg region while the N-terminal extension was estimated to be resolved at between 10 and 11 Å. The resolution estimates for the *T* = 4 VLP were also consistent with the *ResMap* output, measuring between 8 and 10 Å within the core components while the structure of the putative trimeric spike was solved at poorer than 14 Å resolution.

The lower resolution measurements and blurred density for the novel spike within VLPs assembled from the N-terminally extended construct suggest that these features are most likely poorly ordered, limiting our ability to calculate interpretable maps. In addition to the higher degree of disorder seen within these spikes, the local resolution analyses indicated that the *dimeric* spikes formed by HBcAg were also determined to a lower resolution than the capsid shell. This may indicate a degree of flexibility within the HBcAg four-helix bundle or alternatively may be an artefact brought about by the close proximity of the density attributed to the inserted polypeptide. However, close comparison of the two methods for local resolution analysis revealed that regions within the spike formed from HBcAg dimers were differently identified as either high or low resolution (compare Figs. 2C, D and 3C, D) confounding the interpretation of these data. Overall *ResMap* indicates a higher resolution for both reconstructions but also gives a pattern of localised patches of very low-resolution. *Blocres* indicates a more uniform distribution of resolution measurements. In the *T* = 3 structure the shell of the capsid is seen to be higher resolution than the distal portions of the spikes; the base of the four-helix bundle is characterised as being close to 5 Å resolution.

### 3.3 Visualisation of secondary structure elements within the *T* = 3 VLP reconstruction following sharpening

**Fig.4:**
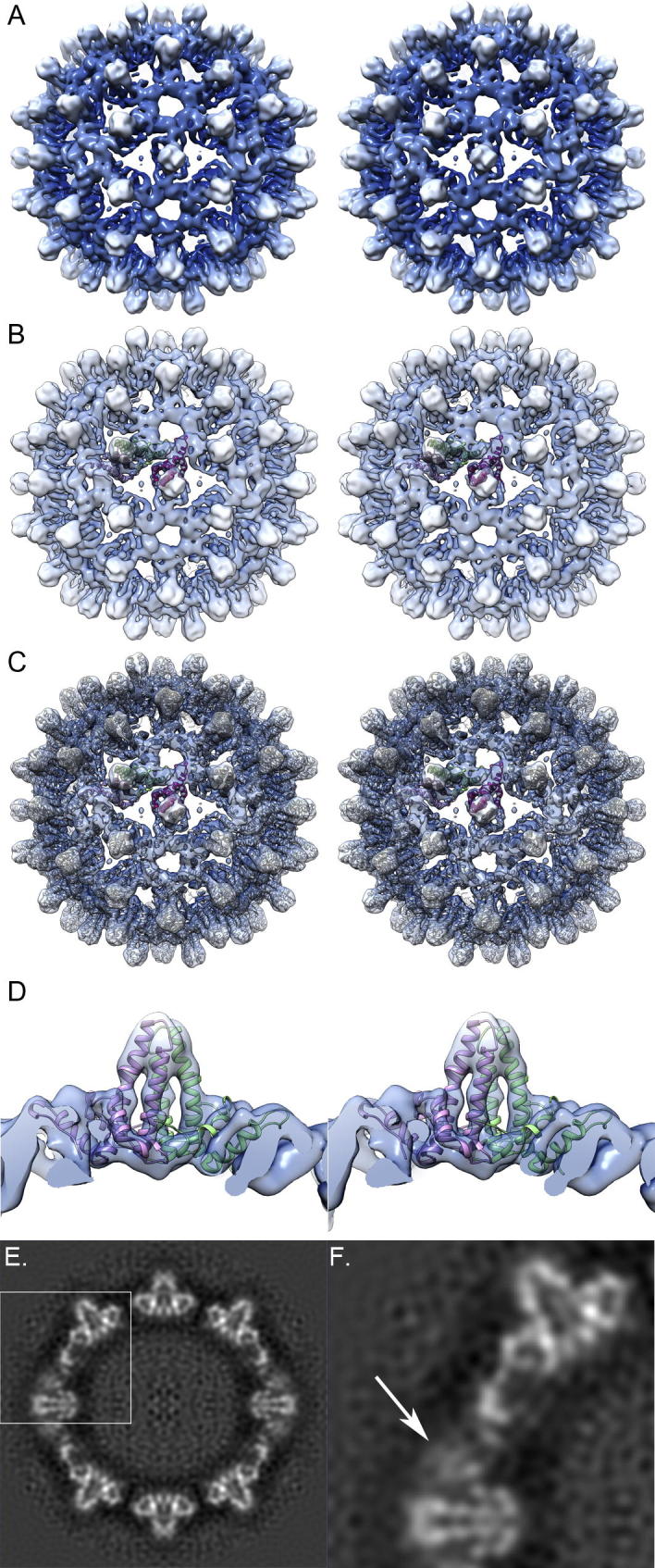
Reconstructions of the sharpened *T* = 3 His-β-L HBcAg VLP shows extensive secondary structure. (A) The *T* = 3 VLP structure is shown sharpened to 8 Å resolution and at an arbitrary isosurface threshold, set to highlight secondary structure elements. (B) The crystal structure of each HBcAg monomer from the *T* = 4 capsid structure (PDB ID 1QGT) was docked to each quasiequivalent position within the *T* = 3 asymmetric unit. Interestingly chain A (mauve), chain B (green) and chain C (magenta) from the *T* = 4 structure each gave the best fit when docked into those respective positions in the *T* = 3 reconstruction. (C) Expanding the symmetry of the asymmetric unit highlights the good fit of the HBcAg crystallographic coordinates into the cryoEM 3D reconstruction. (D) A close up view of a single AB dimer docked into the 3D reconstruction shows that the four-helix bundle is very well resolved. (E and F) Cross-sections through the reconstructed density show that following sharpening the N-terminal spike density is still somewhat blurred. Density measurements show it to be ∼45 percent of the peak intensity value for the map. This suggests that although flexible the feature is most likely constrained by trimerisation.

Local resolution testing appeared to indicate that regions of the *T* = 3 VLP may have been solved at between 6 and 8 Å. To compare our data with the known structures for the *T* = 4 HBcAg particle we sharpened the map by applying a B-factor correction of −488 Å^2^ with a low-pass filter at 8 Å resolution. We then docked individual chains from the X-ray structure of HBcAg into our map. Interestingly the best fit was achieved when chains A, B and C from the X-ray structure 1QGT (the *T* = 4 capsid) were docked into quasiequivalent positions A, B and C respectively in the *T* = 3 reconstruction (Fig. 4). Close inspection of the sharpened reconstruction and docked coordinates shows excellent agreement between the structures and indicates that the secondary structure elements within the *T* = 3 His-β-L HBcAg VLP were well resolved. Unfortunately after sharpening the N-terminal extension remained poorly resolved and at the very high isosurface threshold used to visualise the secondary structure elements in Fig. 4, was not visible.

### 3.4 Prediction of secondary structure elements

**Fig.5:**
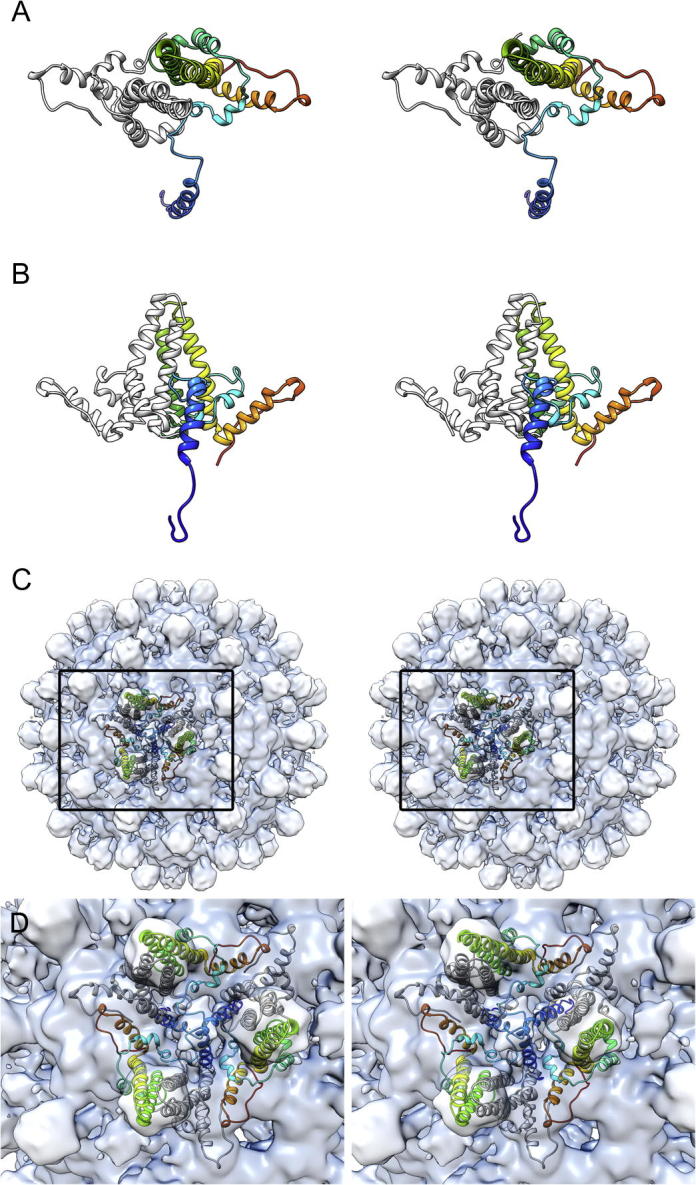
Homology modelling of His-β-L HBcAg. To evaluate the structure of the N-terminal extension of His-β-L HBcAg the sequence was homology modelled in MOE against the known structure of HBcAg with outgap modelling to predict the structure of the extension. The models revealed a largely unstructured polypeptide with regions of α-helix close to the N-terminus. The best model is shown (rainbow – blue = N terminus, red = C terminus) as a stereo pair viewed from the top (A) and side (B) and as a dimer with the HBcAg structure (grey) for comparison. In this model the N-terminus is oriented towards the capsid interior. Most likely this is not correct as this region includes the polyhistidine tract that was employed in purifying the particles by immobilised metal affinity chromatography. Moreover docking the model into our sharpened *T* = 3 reconstruction (thresholded to enclose the correct molecular volume) reveals that the model does not fit into the reconstructed density and there is substantial collision with symmetry related protomers (C and D). Three dimers are shown arranged about the N-terminal spike feature at a local threefold symmetry axis. Each monomer that contributes an N-terminal extension to the spike feature is shown in the rainbow colour scheme, while the symmetry related monomers are shown as the HBcAg structure in grey.

To further understand the structure of the N-terminal extension in His-β-L HBcAg VLPs we used MOE to model the tertiary structure of the protein. The models judged to be the best predicted that the extension would contain an unstructured region followed by an alpha helix located between residues Met12-Asp28, Ser4-Asp24 or His10-Asp28 in the top 3 models. Thus in two models the alpha helix was C-terminal to the polyhistidine tract while the remaining model incorporated the 6HIS motif into the helix. The length of the alpha helix varied in the different models ranging between 24 and 29 Å. Upon comparison with our *T* = 3 structure the models did not match the reconstructed density well, the orientation of the N-terminal extension varied widely between predicted structures and in all cases clashed with symmetry related protomers (Fig. 5). As the reconstructed density for the N-terminal extension was poorly resolved, no attempt was made to refine the models by flexible fitting.

While it is somewhat unsatisfactory to have not been able to produce a robust density map or model of the N-terminal extension and thereby unequivocally demonstrate the trimeric nature of the resultant spike, we conclude that they are indeed trimers. The reconstructed density for these features measured between 55 and 65 percent of the peak intensity within the reconstructions (Fig. 1F and G). Following b-factor correction the density was greater than 45% of the peak value (Fig. 4E and F). This strongly suggests that the spike, although not rigidly ordered is nonetheless constrained. Taken together with our modelling, that predicts a largely disordered polypeptide chain, it therefore seems considerably more likely that the density is the result of a stabilizing interaction between the inserted N-terminal arms than the consequence of three disordered terminal arms extending into the local threefold region being averaged in an incoherent fashion.

## 4 Discussion

We have described the results of a structural analysis of VLPs produced by HBcAg harbouring an N-terminal extension of 37 residues that included a polyhistidine tract. This construct was created to facilitate large-scale production of HBV core protein for diagnostic purposes (Yap et al., 2009, 2010). We were initially interested to investigate the structure of the N-terminal extension to establish the orientation of the polyhistidine tract and thereby confirm its availability for binding to immobilized metal ions in the purification process.

Cryogenic electron microscopy and three-dimensional image reconstruction were used to characterise the VLPs at intermediate resolution. The resultant preliminary reconstructions revealed the presence of an additional spike located at sites of threefold symmetry where the N-terminus of the HBcAg emerges from the capsid surface.

Efforts to determine high-resolution structure data for this feature were unsuccessful owing to the presence of a degree of disorder. The unexpected presence of a second spike, thought to be formed by trimerisation of the N-terminal extension, led us to hypothesise that this site may be suitable for multivalent display of antigens that natively form trimers. We therefore sought to better understand the structure of the novel spike feature by extending the resolution of our reconstructions. However, despite a fivefold increase in the dataset there was no increase in resolution. Published structures of the HBcAg solved by cryo-EM have achieved resolutions up to 3.5 Ǻ (Yu et al., 2013). Thus we thought it most unlikely that our failure to achieve high-resolution related to a feature of the HBcAg that was limiting the resolution. The poor resolution of the reconstructions was therefore attributed to the presence of the N-terminal extension. To confirm this hypothesis, we performed experiments to evaluate local resolution within the maps. These tests confirmed our view, yielding lower resolution measurements for the inserted polypeptide than for the HBcAg component.

We propose that the lower resolution of the insert is due to it being more flexible than the rest of the capsid. Aside from a single α-helix, most of the insert sequence is not predicted to form any particular structural motifs, supporting the assertion that it is more flexible than the HBcAg component. Our local resolution analyses suggested that the dimer spikes of the HBcAg component were also less well resolved than the floor of the particle; *Blocres* indicates 10 Å in the distal spike region of the *T* = 3 VLP while measurements close to 5 Å are seen at the base. In our sharpened *T* = 3 map however this region is clearly seen and closely matches the features of the docked coordinates of the HBcAg X-ray structure when low-pass filtered at 8 Å resolution (Fig. 4D). We conclude then that the flexibility of the insert region may have affected resolution measurements for these 4-helix bundles. The distal portion of the HBcAg spikes was evaluated as having the lowest resolution of the core component, however the region comprises robust secondary structure and loop regions are comparatively short. In our reconstructions the inserted polypeptide’s density is in close proximity to the dimer spike on one side (Fig. 1). This may therefore compromise resolution measurements, in particular the local FSC approach of *Blocres*, which measures the extent of agreement between maps calculated from half datasets using a 30 voxel^3^ sliding box. Moreover, the flexible region may introduce an uncertainty in the alignment of the projection images, degrading the resolution of the entire reconstruction.

Disorder is a perennial problem in structural biology, frequently preventing crystallogenesis or leading to unresolvable features in X-ray studies. For cryoEM experiments, disorder compromises achievable resolutions and yields blurred density. Our analysis clearly illustrates the value of local-resolution assessment in such cases. In this study we might have concluded on the basis of FSC^0.5^ resolution assessment that we had not achieved sufficient resolution to make secondary structure determination possible. In this case the higher-resolution features of our map were of an already well-characterised protein (HBcAg). In similar studies of unknown structures however, where the protein structure is highly ordered in places but disordered in others, a single resolution measurement may lead to an overly pessimistic interpretation. We conclude then that local-resolution assessment should be routinely included in cryoEM workflows.

Despite efforts to attain high-resolution, our reconstructions did not reveal the location of the N-terminus of the extended protein. The VLPs were however purified by metal affinity chromatography. Thus the 6-His tract, which is located 4 amino acids from the N-terminus is at least surface accessible. We cannot unfortunately ascertain whether the N-terminus is in a favourable orientation to act directly as a platform for further extensions. The flexibility of the insert is potentially advantageous however, possibly reducing the constraints on the positioning of the termini, similar to the manner in which glycine linkers facilitate insertions into the MIE loop (Nassal et al., 2008). The trimeric conformation and outward orientation of the insert is encouraging and suggests future opportunities for the introduction of heterologous sequences, particularly if the inserts are themselves capable of interacting to form a trimer. For example many viral surface proteins are trimeric in nature. Thus work to develop N-terminal extensions of HBcAg for multivalent display of heterologous proteins that natively form trimers might prove to be a fruitful line of research for future vaccine development.
